# In Situ Monitoring of Non-Thermal Plasma Cleaning of Surfactant Encapsulated Nanoparticles

**DOI:** 10.3390/nano14030290

**Published:** 2024-01-31

**Authors:** Gengnan Li, Dmitri N. Zakharov, Sayantani Sikder, Yixin Xu, Xiao Tong, Panagiotis Dimitrakellis, Jorge Anibal Boscoboinik

**Affiliations:** 1 Center for Functional Nanomaterials, Brookhaven National Laboratory, Upton, NY 11973, USA; dzakharov@bnl.gov (D.N.Z.); sayantani.sikder@stonybrook.edu (S.S.); 1xuyixin@163.com (Y.X.); xtong@bnl.gov (X.T.); 2 Department of Materials Science and Chemical Engineering, Stony Brook University, Stony Brook, NY 11790, USA; 3 Catalysis Center for Energy Innovation, Department of Chemical and Biomolecular Engineering, University of Delaware, Newark, DE 19716, USA; p.dimitrakellis@certh.gr

**Keywords:** non-thermal plasma treatment, in situ spectroscopy, environmental transmission electron microscopy, surfactant, nanoparticles

## Abstract

Surfactants are widely used in the synthesis of nanoparticles, as they have a remarkable ability to direct their growth to obtain well-defined shapes and sizes. However, their post-synthesis removal is a challenge, and the methods used often result in morphological changes that defeat the purpose of the initial controlled growth. Moreover, after the removal of surfactants, the highly active surfaces of nanomaterials may undergo structural reconstruction by exposure to a different environment. Thus, ex situ characterization after air exposure may not reflect the effect of the cleaning methods. Here, combining X-ray photoelectron spectroscopy, in situ infrared reflection absorption spectroscopy, and environmental transmission electron microscopy measurements with CO probe experiments, we investigated different surfactant-removal methods to produce clean metallic Pt nanoparticles from surfactant-encapsulated ones. It was demonstrated that both ultraviolet-ozone (UV-ozone) treatment and room temperature O_2_ plasma treatment led to the formation of Pt oxides on the surface after the removal of the surfactant. On the other hand, when H_2_ was used for plasma treatment, both the Pt^0^ oxidation state and nanoparticle size distribution were preserved. In addition, H_2_ plasma treatment can reduce Pt oxides after O_2_-based treatments, resulting in metallic nanoparticles with clean surfaces. These findings provide a better understanding of the various options for surfactant removal from metal nanoparticles and point toward non-thermal plasmas as the best route if the integrity of the nanoparticle needs to be preserved.

## 1. Introduction

Synthesis of metal nanoparticles is of great importance in both fundamental studies and practical applications [1,2,3,4,5], such as heterogeneous catalysis [6,7,8,9], drug delivery [10,11], quantum dots [12], electronic devices, and purification systems [13,14,15]. A common strategy used in wet synthesis to control nanoparticle size is adding surfactants [16,17,18,19]. As a result, the nanoparticle surface is decorated by surfactant molecules, which prevent particle aggregation and thus stabilize the particles at the nanoscale. Generally, the surfactants used in synthesis include alkyl-based molecules, peptides, lipids, deoxyribonucleic acid (DNA), and polymers covalently grafted to or non-covalently assembled on nanoparticle surfaces, thereby changing their properties [6]. For example, when organic ligands are used, the size of nanoparticles can be controlled, and the shape of nanoparticles can be regulated [20,21,22,23,24,25]. The different atomic arrangements on crystal surfaces can affect catalytic behavior in terms of activity, selectivity, and durability. However, for most applications, the surfactant layer needs to be removed post-synthesis; this is true for, for example, in catalysis, where the surface sites need to be accessible to reactants, and in biomedical applications, due to the high cytotoxicity of the surfactants [26,27,28,29].

Thermal treatment is one of the most efficient strategies for removing hydrocarbons [30]. However, the decomposition of hydrocarbons requires a relatively high temperature, which may induce changes in the structure and surface composition of metal nanoparticles. Therefore, efforts have been made to develop cleaning strategies to remove the surfactant layer under non-thermal conditions. Among various approaches, ultraviolet-ozone (UV-ozone) [31,32] and oxygen (O_2_) plasma treatment [26,33,34,35] have been widely studied. The UV-ozone process involves the simultaneous UV-photon-induced activation of the C–H bonds (photosensitized process) and the oxidation by atomic oxygen O species derived from O_3_, resulting eventually in the decomposition of organic surfactants into carbon dioxide (CO_2_) and water (H_2_O). O_2_ plasma is abundant in highly reactive O species that participate in rapid oxidative degradation of organic species towards CO_2_ and H_2_O. Recently, atmospheric pressure helium (He)/O_2_ plasma was successfully applied to remove organic ligands and enhance the catalytic performance of supported palladium (Pd) nanocubes [36].

For both strategies, a clean surface can be obtained upon desorption of CO_2_ and H_2_O. Nevertheless, the oxidation reactions between oxygen species and surface metal atoms are inevitable, which lead to forming an oxide layer/cluster on the nanoparticles’ surface, and thus sequential reduction steps are required. To avoid the oxidation of metal nanoparticles, inert gases, such as argon or helium, have been used for plasma treatment [37]. In addition, the non-oxidative plasma treatments are ex situ operations (i.e., the sample is characterized after treatment rather than during). After plasma treatment, the sample is exposed to air. Thus, the highly active atoms on metal nanoparticles can react with airborne species (i.e., H_2_O, O_2_, hydrocarbons), which may lead to the modification of the active surface. It becomes inevitable that multiple reaction steps during surfactant layer removal result in structural transformations directly affecting the surface chemistry of metal nanoparticles. Thermal reduction treatments are expected to make those transformations even more prominent. Therefore, it is necessary to study nanoparticle structural transformations during the treatment (in situ). However, ex situ characterization techniques are poorly suited for this purpose as they do not necessarily reflect the actual modifications after particles are transferred between treatment and characterization chambers through air.

Recent developments in the field of in situ characterization (i.e., at relevant conditions) provide a unique opportunity to monitor structural changes of materials during non-thermal plasma treatment. For example, de Mello and co-workers [38] reported the in situ plasma treatment of metal-organic-framework (MOF) films using different gases. Infrared reflection absorption spectroscopy (IRRAS) shows that the O_2_ plasma treatment leads to the etching of organic ligands with the formation of carbonyl groups. In contrast, N_2_ plasma treatment induces mild etching and formation of nitrile groups on the MOF. Combining organic ligand cleavage and functionalization in that work results in enhanced selectivity in gas permeation using MOF-based membranes. More importantly, plasma treatment coupled with simultaneous spectroscopic measurements (in situ) at different conditions enables the fundamental understanding of surface modification by plasma treatment, eliminating the uncertainty of exposure to uncontrolled environments between the treatment and characterization steps (ex situ characterization).

In this work, surfactant-encapsulated platinum (Pt) nanoparticles have been synthesized through a chemical reduction method using cetyltrimethylammonium bromide (CTAB) and oleylamine as protecting agents. We characterize the surface modification of metal nanoparticles using a combination of in situ spectroscopic measurements to illustrate the mechanistic aspects of surface cleaning by non-thermal plasma treatment in different gases, such as O_2_, H_2,_ and sequential O_2_–H_2_. Ex situ UV-ozone treatment on surfactant-encapsulated Pt nanoparticles is also studied for comparison. Environmental TEM (ETEM) shows the efficient removal of surfactants by plasma treatment. X-ray photoelectron spectroscopy (XPS) coupled with in situ IRRAS has been applied to quantitively understand the effect of different gases in plasma treatment on the structure and properties of Pt nanoparticles. The effectiveness of surfactant removal treatments has been investigated by in situ carbon monoxide (CO) probe studies. This work demonstrates the profound effects of gases used in plasma treatment on the surface cleaning of metal nanoparticles. These observations may open opportunities for the utilization of nanoparticles in catalysis, surface science, and biomedical applications that require efficient removal of surfactants by reaching a better mechanistic understanding of this process and identifying the most adequate conditions.

## 2. Materials and Methods

### 2.1. Materials Synthesis

Gold (Au, Sigma-Aldrich, St. Louis, MI, USA, 99.99+%)-coated silicon (Si) substrates were prepared by physical vapor deposition on a Kurt J. Lesker PVD75 system (Lesker Company, Jefferson Hills, PA, USA). To enhance the interaction between Au and Si, a 10 nm adhesive layer of titanium (Ti, Sigma-Aldrich, 99.99%) was first deposited on Si, with a deposition rate of 0.5 Å/s. Then, a 100 nm Au layer was deposited onto the Ti-Si substrate with a deposition rate of 1.0 Å/s. The obtained Au substrate was analyzed by atomic force microscopy (AFM) on a Park NX20 atomic force microscopy.

Surfactant-encapsulated Pt nanoparticles were synthesized by a chemical reduction method [39]. In a typical synthesis, 2.5 mg chloroplatinic acid hexahydrate (H_2_PtCl_6_·6H_2_O, Sigma-Aldrich, ACS reagent) and 20.0 mg hexadecyltrimethylammonium bromide (CTAB, Sigma-Aldrich, BioXtra, >99%) were mixed in 4.5 mL deionized (DI) water. The solution was heated to 50 °C under constant stirring (400 rpm) for 2 h. Then, 3.0 mg sodium borohydride (NaBH_4_, Sigma-Aldrich, ReagentPlus, 99%) was dissolved in 0.5 mL ice-cooled DI water and added dropwise into the solution. The resulting mixture was stirred for another 12 h at 50 °C. The Pt nanoparticles were purified by discarding the precipitate following centrifugation at 3000 rpm for 30 min. The procedure was repeated four times.

To obtain oleylamine-encapsulated Pt nanoparticles, CTAB encapsulated Pt nanoparticles were collected by centrifugation at 13,000 rpm for 30 min. The resulting precipitate was washed with DI water twice and redispersed in oleylamine (Sigma-Aldrich, >98% primary amine)-water solution (0.8 mL oleylamine in 1 mL DI water). The suspension in a closed container was heated to 50 °C and stirred for 12 h. The obtained oleyamine encapsulated Pt nanoparticles were washed three times with methanol (anhydrous, Sigma-Aldrich, 99.8%) and then redispersed in 0.5 mL toluene (anhydrous, Sigma-Aldrich, 99.8%).

The solution was slowly dropped onto a water subphase on a Langmuir-Blodget trough to produce a monolayer of oleylamine-encapsulated Pt nanoparticles. After evaporation of the toluene for 1 h, the film was compressed until a surface tension of 15 mN·m^−1^ was achieved. The resulting film was then aged for 30 min before being transferred to an Au-coated Si substrate via a pull-out method.

### 2.2. Structural Characterization

Scanning electron microscopy (SEM) was performed on a Hitachi 4800 SEM instrument (Hitachi High-Technologies Corporation, Chiyoda-Ku, Tokyo, Japan), operating at 10 kV. Environmental transmission electron microscopy (ETEM) was performed on aberration-corrected FEI Titan 80–300 (S)TEM instrument (FEI Company, Hillsboro, OR, USA) operated at 300 kV.

Ambient-pressure X-ray photoelectron spectroscopy (XPS) analysis was performed using a customized system, with a Hemispherical Energy Analyzer PHOIBOS NAP 150 (SPECS Surface Nano Analysis GmbH, Berlin, Germany), with monochromatic Al Kα as the excitation source (hν = 1486.6 eV) at Center for Functional Nanomaterials at Brookhaven National Laboratory. The base pressure was 2 × 10^−9^ mbar. The spectra regions probed included C 1s, O 1s, N 1s, Au 4f, and Pt 4f. For each spectral region, a pass energy of 20 eV and energy step size of 0.1 eV were used. Peak fitting was performed using CasaXPS (Version 2.3.24PR1.0) peak fitting software. A Shirley background type was used. Lineshapes (1.2, 85, 70) and GL (30) were used for Pt^0^ and Pt^δ+^ peak fitting, respectively.

Infrared reflection absorption spectroscopy (IRRAS) spectra were collected on a Bruker Vertex 80 V spectrometer equipped with a mercury-cadmium-telluride (MCT) detector with a grazing incidence angle of 8°. The base pressure of IRRAS was 2 × 10^−8^ mbar. An average of 1000 scans were collected over a range of 800–4000 cm^−1^ with a resolution of 4 cm^−1^ after a certain time of plasma treatment. The gas pressure (O_2_ or H_2_) was maintained at 0.1 mbar. In all cases, both the *p*- and *s*-polarized light spectra were taken under all conditions. The $\frac{p_{0}}{s_{0}}$ of the starting material at 0.1 mbar gas pressure was used as a reference. The spectra taken at different plasma treatment time were calculated by comparing to the reference: $\frac{\frac{p}{s}}{\frac{p_{0}}{s_{0}}}$.

Likewise, for in situ CO probe experiment, the $\frac{p_{0}}{s_{0}}$ of starting material at 2 × 10^−8^ mbar was used as reference. By changing the pressure of CO in the IRRAS chamber, the spectra of CO adsorption on Pt nanoparticles were collected and converted to transmission mode by comparing to the reference: $\frac{\frac{p}{s}}{\frac{p_{0}}{s_{0}}}$.

### 2.3. Plasma and UV-Ozone Treatments

For plasma treatment setup on the IRRAS instrument, the setup description is shown in Scheme S1 in the supporting information [38]. All the plasma treatments were performed at room temperature (25 °C). First, the gas of interest was introduced into the IRRAS chamber to a pressure of 0.1 mbar for all plasma treatment conditions. Then, the plasma was generated using an AC high-voltage at 1 kV peak-to-peak with a frequency of 22 kHz (PVM500, Information Unlimited, Mont Vernon, NH, USA). The voltage and current waveforms were recorded using a high-voltage probe (Tektronix P6015 (Tektronix, Inc., Beaverton, OR, USA)) and a current monitor (Pearson 6585 (Pearson Electronics, Inc. Palo Alto, CA, USA)), respectively. Both signals were observed in real-time using a Tektronix MDO32 series oscilloscope, and the average power was calculated by integrating the instantaneous power over one period. The plasma was characterized using optical emission spectroscopy (OES). The plasma emission was collected through an optical fiber with a collimating lens adjusted to a quartz viewport of the reactor chamber. The wideband spectrum was recorded using an AvaSpec-ULS4096CL-EVO (Avantes BV, Apeldoorn, The Netherlands) spectrometer and analyzed with AvaSoft8.14.0.0 software.

For UV-ozone treatment, the Pt nanoparticles on Au film were placed in a UV-ozone cleaning system (UVOCS INC., Lansdale, PA, USA; model: T10 × 10/OES) and subject to UV irradiation (185 nm and 254 nm) for 30 min.

## 3. Results and Discussion

In the following subsections, we will present the spectroscopy results for surfactant removal using different methods. Namely, Section 3.1 will focus on UV-ozone treatment, Section 3.2 on oxygen plasma, Section 3.3 on hydrogen plasma, and Section 3.4 on a combination of UV-ozone or oxygen plasma, followed by hydrogen treatments (thermal or plasma) to reduce the produced oxides.

### 3.1. Removal of Surfactant by UV-Ozone Treatment

As described in the experimental section, surfactant-encapsulated Pt nanoparticles were prepared using methods from the literature with CTAB and chloroplatinic acid as precursors (H_2_PtCl_6_) [39,40]. A thin film of Pt nanoparticles was prepared by the Langmuir-Blodgett (LB) method and then transferred onto a Au-coated silicon wafer. The original capping agent CTAB was exchanged to oleylamine to fabricate the LB assembly and deposition. For comparison, the surfactant-encapsulated Pt nanoparticles were first cleaned by UV-ozone treatment. X-ray photoelectron spectroscopy (XPS) was performed to track surface composition changes. The survey spectrum of the fresh sample in Figure S1 (supporting information) shows that the surface mainly contains carbon (C), gold (Au), platinum (Pt), and oxygen (O). The oxidation state of Au remains unchanged throughout all the experiments.

**Figure 1:**
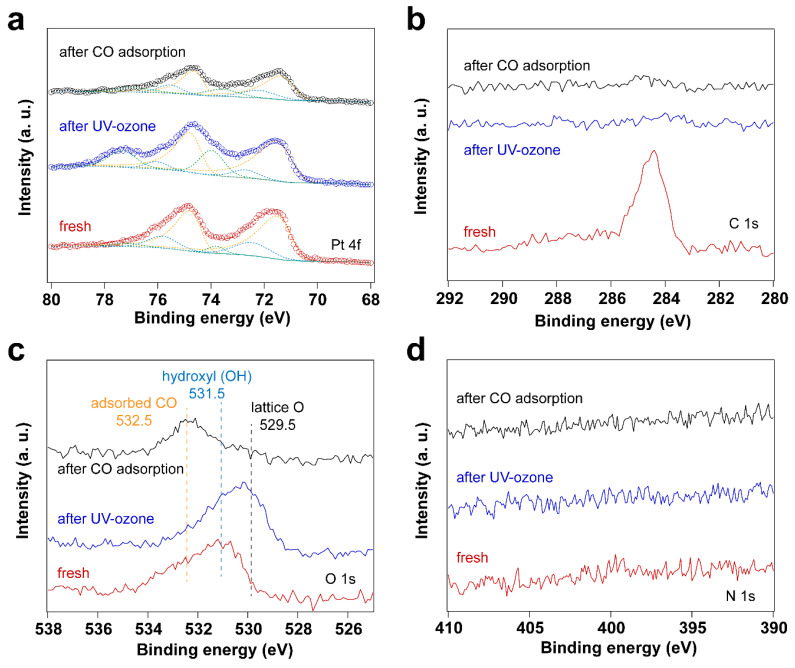
XP spectra of surfactant encapsulated Pt nanoparticles before and after 30 min UV-ozone treatment and after subsequent exposure to 10 mbar of CO at room temperature: (**a**) Pt 4f XP spectra; (**b**) C 1s XP spectra; (**c**) O 1s XP spectra, (**d**) N 1s XP spectra.

**Figure 2:**
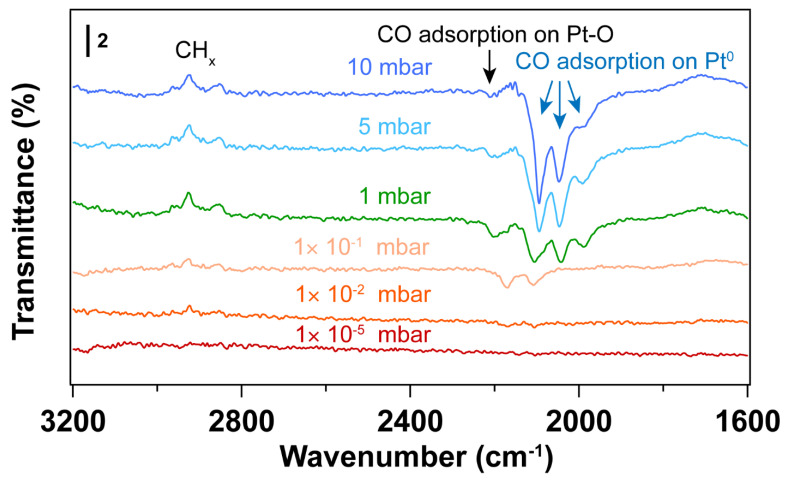
IRRA spectra of in situ CO adsorption on Pt nanoparticles after UV-ozone treatment. Before introducing CO, background ($\frac{p_{0}}{s_{0}}$) was collected under UHV conditions (2 × 10^−8^ mbar); The transmittance signal was obtained by comparing the spectra under different CO pressures with the background spectrum ($\frac{{p_{CO}/s}_{CO}}{{p_{0}/s}_{0}}$); where *p_CO_* and *s_CO_* refer to the *p*- and *s*- polarized spectra that were collected under different CO pressures. The wavenumbers of CO vibration are presented in brackets.

Since the surfactant molecules, oleylamine and CTAB, contain hydrocarbon CH_x_ and amine (NH_2_) groups, both C and N are detected by C 1s and N 1s for the fresh sample. After UV-ozone treatment, the peak of C 1s disappears, which is also reflected by the high-resolution C 1s and N 1s spectra. As shown in Figure 1, the disappearance of C 1s and N 1s peaks indicates that the surfactant molecules were removed after UV-ozone treatment. The Pt 4f spectra displayed in Figure 1 demonstrate Pt oxidation after UV-ozone treatment, as evidenced by the appearance of peaks corresponding to Pt–O species [41,42,43,44]. Table 1 summarizes the peak positions and the ratio of Pt–O/Pt^0^. Two different Pt–O species were observed and assigned to Pt(II)–O and Pt(I)–O. It shows a larger fraction of surface Pt–O(II) than Pt–O(I) after UV-ozone treatment, suggesting the significant oxidation of Pt. Therefore, when in situ IRRAS CO probe experiments were performed, CO molecules adsorb not only on metallic Pt (Pt^0^) sites, but also on Pt–O sites, as shown in Figure 2. Correspondingly, different O species, such as lattice O and adsorbed O, are observed in the O 1s spectra after UV-ozone treatment due to the reaction between active O species generated from UV-ozone and the organic surfactant [45,46].

As shown in Figure 2, the CO vibrational frequencies at 2200–2100 cm^−1^ in IRRAS are characteristic CO adsorption on oxidized Pt sites [47,48], while the bands in the 2100–1900 cm^−1^ range correspond to CO molecules linearly adsorbed on Pt nanoparticles (atop) [49,50,51]. The peak areas for the CO vibrational mode corresponding to metallic Pt sites increase with increasing pressure, while the peak area corresponding to CO on Pt-O sites decreases, suggesting the reduction of Pt–O during CO exposure. As confirmed by the Pt 4f spectra (Figure 1), metallic Pt is dominant and only a small fraction of Pt–O(I) was observed after CO exposure to elevated pressures. In the presence of CO, the Pt–O(II) was first reduced to Pt–O(I), followed by the reduction to Pt^0^. Therefore, different CO vibrational frequencies were observed due to the different oxidation states of Pt in the presence of CO.

At 1 × 10^−5^~1 × 10^−1^ mbar CO pressure range, CO initially adsorbs on Pt–O sites with vibrational frequency of ~2170 and 2110 cm^−1^ (See Figure S2 in the supporting information) [48]. Further increasing the CO pressure, CO adsorption on metallic Pt sites emerge. When the pressure of CO reaches 5 mbar, atop adsorption of CO on metallic Pt sites is dominant, while the CO adsorption on Pt–O sites almost disappears. This likely indicates reduction of the Pt oxides by CO. It is well known that the differences in CO stretching frequency can arise from the different sites on Pt nanoparticles. That is, the undercoordinated sites lead to stronger CO adsorption than high-coordination sites, resulting in lower CO vibrational frequencies [47]. Consequently, the bands at ~2048 and ~1992 cm^−1^ that appeared at a CO pressure of 1 mbar can be assigned to the different metallic Pt sites. Further increasing CO pressure, CO adsorption on higher coordination sites of metallic Pt (~2096 cm^−1^) is obtained [47]. Moreover, the desorption of hydrocarbon species (as evident by the C-H stretching vibrations at 3000–2800 cm^−1^) was observed with the increase of CO pressure, indicating the presence of hydrocarbon residuals on the Pt surface after UV-ozone cleaning. Note that these appear as peaks pointing up in the transmittance spectra in Figure 2. This result is also consistent with the decreasing peak area ratio of C 1s/Au 4f after in situ CO probe experiment (Table S1 in supporting information).

### 3.2. Removal of Surfactant by O_2_ Plasma Treatment

**Figure 3:**
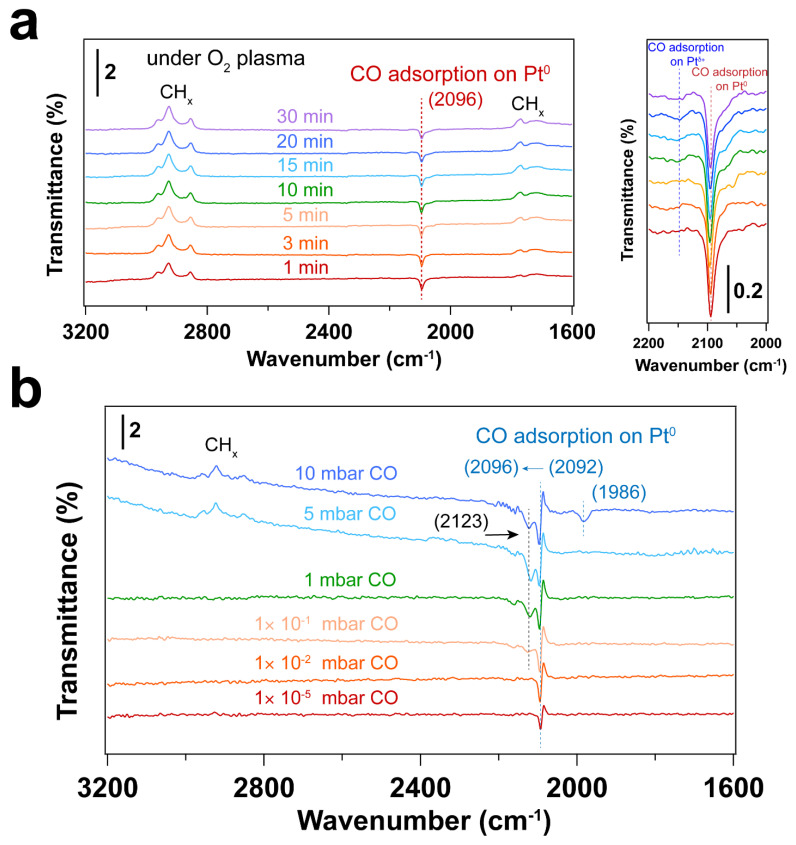
(**a**) IRRA spectra of surfactant encapsulated Pt nanoparticles during O_2_ plasma treatment at room temperature. The CO adsorption range (2200–2000 cm^−1^) is also presented. The pressure of O_2_ was 0.1 mbar, and the power applied for plasma treatment was 4 W. Before plasma treatment, background ($\frac{p_{0}}{s_{0}}$) was collected in the presence of 0.1 mbar O_2_. The transmittance signal was obtained by comparing the spectrum after plasma treatment with the background spectrum ($\frac{{p_{t}/s}_{t}}{{p_{0}/s}_{0}}$); where *p_t_* and *s_t_* refer to the *p*- and *s*- polarized spectra; (**b**) IRRAS spectra of in situ CO adsorption on Pt nanoparticles after O_2_ plasma treatment. Before introducing CO, background ($\frac{p_{0}}{s_{0}}$) was collected under UHV conditions (2 × 10^−8^ mbar). The transmittance signal was obtained by comparing the spectra under different CO pressures with the background spectrum ($\frac{{p_{CO}/s}_{CO}}{{p_{0}/s}_{0}}$), where *p_CO_* and *s_CO_* refer to the *p*- and *s*-polarized spectra collected under different CO pressures. The wavenumbers of CO vibration are presented in brackets.

**Figure 4:**
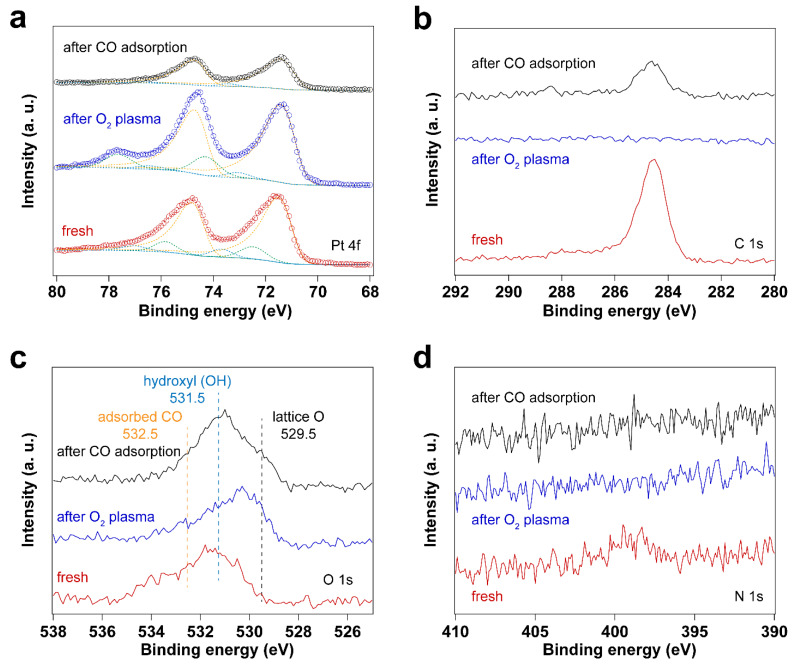
XP spectra of surfactant encapsulated Pt nanoparticles before and after O_2_ plasma treatment and after in situ CO adsorption experiment: (**a**) Pt 4f XP spectra; (**b**) C 1s XP spectra; (**c**) O 1s XP spectra; (**d**) N 1s XP spectra.

Figure 3 and Figure 4 display IRRAS and XPS measurements of surfactant-encapsulated Pt nanoparticles cleaned by in situ O_2_ plasma treatment. Similar to the UV-ozone cleaning process, carbon species on the surface can be efficiently removed by O_2_ plasma, while Pt was oxidized by the highly active O species generated in the plasma. As shown in Figure 3a, the bands in the 3100–2800 cm^−1^, 2200–1900 cm^−1^ and 1800–1650 cm^−1^ ranges are C–H stretching of the hydrocarbons in the surfactant [52,53], CO adsorption on Pt nanoparticles [47] and C–H bending of hydrocarbons [52], respectively. After 1 min O_2_ plasma treatment, the positive hydrocarbon peaks at 3100–2800 and 1800–1650 cm^−1^ indicate the removal of hydrocarbon species (C–H). During plasma treatment, the active O species react with hydrocarbons, forming CO, which adsorbs on Pt nanoparticles. Note that the presence of CO is not from direct introduction to the chamber but rather the result of the oxidation of hydrocarbons under O_2_ plasma. As shown in Figure 3a, the CO adsorption on Pt^δ+^ sites also suggests that the surface of Pt nanoparticles is oxidized during the O_2_ plasma treatment, and the fraction of Pt–O species increases, which is consistent with the Pt 4f and O 1s spectra as shown in Figure 4. Compared to the UV-ozone treatment, O_2_ plasma treatment results in a smaller fraction of Pt oxide sites on the Pt nanoparticle surface (see Table 2). Only adsorption of CO on metallic Pt sites is observed at CO pressures < 1 × 10^−1^ mbar as evident by the peak originally at 2092 cm^−1^ (in the background) that shifts to 2096 cm^−1^ under CO pressure in Figure 3b, and only a small fraction of Pt–O species (peak at 2123 cm^−1^) is evident at higher pressures. In addition, below 10 mbar of CO, the absence of CO vibrational frequency at lower wavenumber range (2050–1990 cm^−1^) may suggest that less defective sites (low coordination) are formed after the O_2_ plasma treatment in comparison to the UV-ozone process [40]. Further increase of CO pressure to 10 mbar results in additional surge in CO adsorption at lower coordination sites (1986 cm^−1^), which may be due to the structural reconstruction of Pt nanoparticles caused by CO adsorption [54].

### 3.3. Removal of Surfactant by H_2_ Plasma Treatment

**Figure 5:**
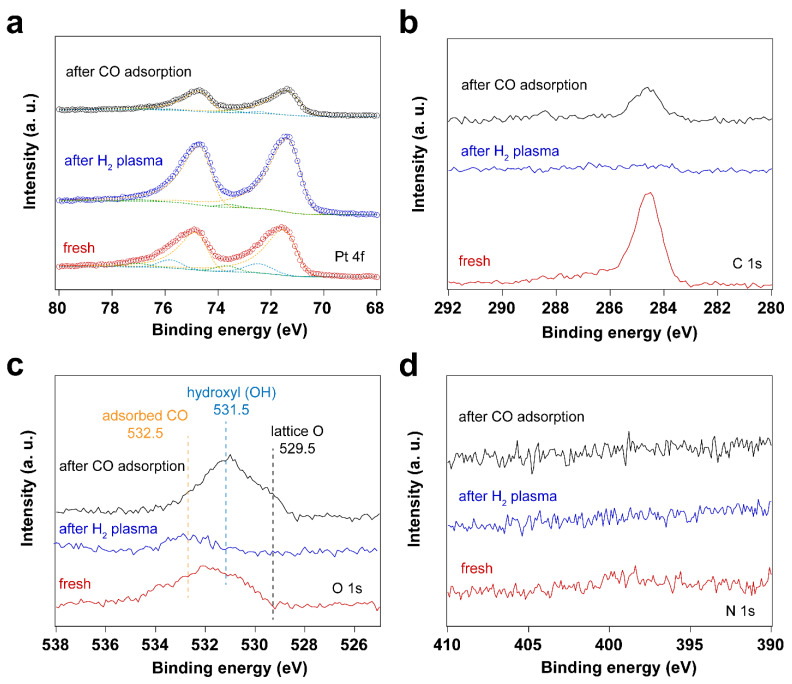
XP spectra of surfactant encapsulated Pt nanoparticles before and after H_2_ plasma treatment and after in situ CO adsorption experiment: (**a**) Pt 4f XP spectra; (**b**) C 1s XP spectra; (**c**) O 1s XP spectra; (**d**) N 1s XP spectra.

Altogether, both UV-ozone and O_2_ plasma treatments change the oxidation states of Pt. At the same time, the surfactant is removed efficiently by forming CO_2_ and CO species, with the latter (CO) adsorbing on the Pt nanoparticles. To avoid the oxidation of the Pt nanoparticles during cleaning, H_2_ plasma treatment was further investigated. Figure 5 shows XP spectra of the fresh sample after H_2_ plasma treatment and after CO adsorption. The data shows that the surfactant can also be removed by H_2_ plasma, as demonstrated by the disappearance C 1s and N 1s peaks in XP spectra. Similar to the sample after O_2_ plasma treatment, C species emerge in C 1s spectrum after CO adsorption experiment on the H_2_ plasma cleaned Pt nanoparticles.

**Figure 6:**
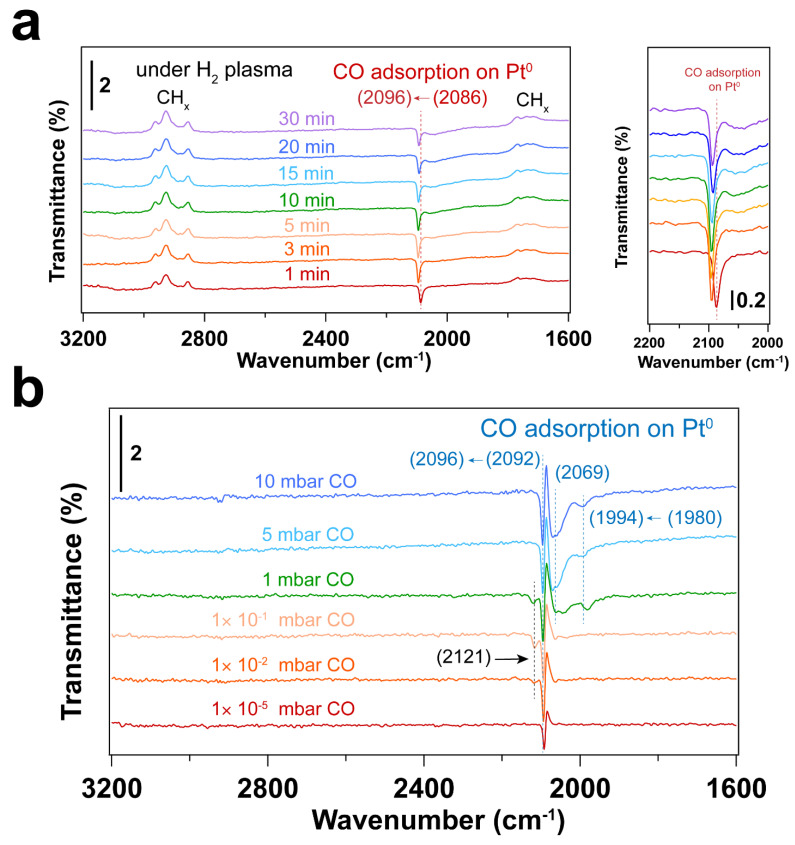
(**a**) IRRA spectra of surfactant encapsulated Pt nanoparticles during H_2_ plasma treatment at room temperature. The CO adsorption range (2200–2000 cm^−1^) is also presented. The pressure of H_2_ was 0.1 mbar, power applied for plasma treatment was 4 W; before plasma treatment, background ($\frac{p_{0}}{s_{0}}$) was collected in the presence of 0.1 mbar H_2_; The transmittance signal was obtained by comparing the spectrum after plasma treatment with the background spectrum ($\frac{{p_{t}/s}_{t}}{{p_{0}/s}_{0}}$); where *p_t_* and *s_t_* refer to the *p*- and *s*- polarized spectra; (**b**) IRRAS spectra of in situ CO adsorption on Pt nanoparticles after H_2_ plasma treatment. Before introducing CO, background ($\frac{p_{0}}{s_{0}}$) was collected under UHV conditions (2 × 10^−8^ mbar); The transmittance signal was obtained by comparing the spectra under different CO pressures with the background spectrum ($\frac{{p_{CO}/s}_{CO}}{{p_{0}/s}_{0}}$); where *p_CO_* and *s_CO_* refer to the *p*- and *s*- polarized spectra that were collected under different CO pressures. The wavenumbers of CO vibration are presented in brackets.

The IRRA spectra in Figure 6a show CO adsorbed on metallic Pt during the H_2_ plasma treatment. This could be due to the reaction between hydrocarbon and surface oxygen species, forming CO. As a result, Pt remains in the metallic state after H_2_ plasma treatment (see Figure 5a and Table 3). Only linear adsorption of CO on Pt^0^ is observed from in situ CO probe experiments, as shown in Figure 6b. Different from the O_2_-based treatment, no CO adsorption on low coordination Pt sites is detected at low CO pressures (<1 mbar) in this case. By increasing CO pressure to 1 mbar, different CO stretching frequencies are obtained. This could be ascribed to the structural reconstruction of surface Pt atoms during CO adsorption [54]. CO initially adsorbs on those high-coordination Pt sites, resulting in CO stretching frequencies of 2069 and 2092 cm^−1^. With increasing CO pressure, the coordination number of Pt atoms varies with the plasma treatment time. Therefore, CO adsorption on low-coordination Pt sites appears (1980 cm^−1^). At high CO pressure, the increased CO coverage on Pt nanoparticles also results in the blue shifting of CO vibrational frequencies, which is caused by dipole-dipole interactions (from 1980 to 1994 cm^−1^ and from 2092 to 2096 cm^−1^). These results demonstrate that H_2_ plasma treatment is an efficient strategy for removing hydrocarbon-based surfactants while preventing oxidation of Pt.

### 3.4. Using H_2_ Plasma to Reduce Pt Nanoparticles Treated by O_2_ Sources

**Figure 7:**
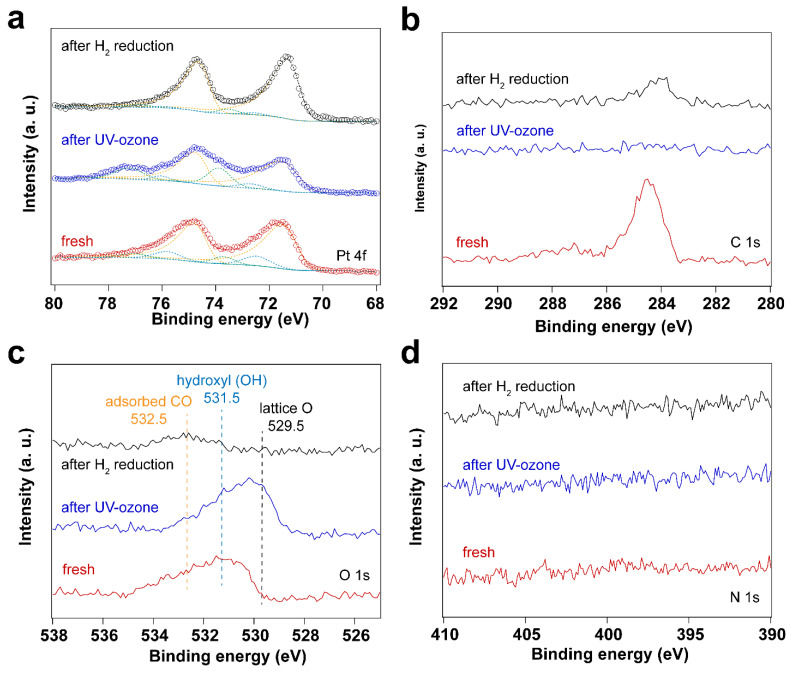
XP spectra of surfactant encapsulated Pt nanoparticles after UV-ozone treatment followed by in situ H_2_ reduction at 100 °C under 0.5 mbar H_2_: (**a**) Pt 4f XP spectra; (**b**) C 1s XP spectra; (**c**) O 1s XP spectra; (**d**) N 1s XP spectra.

**Figure 8:**
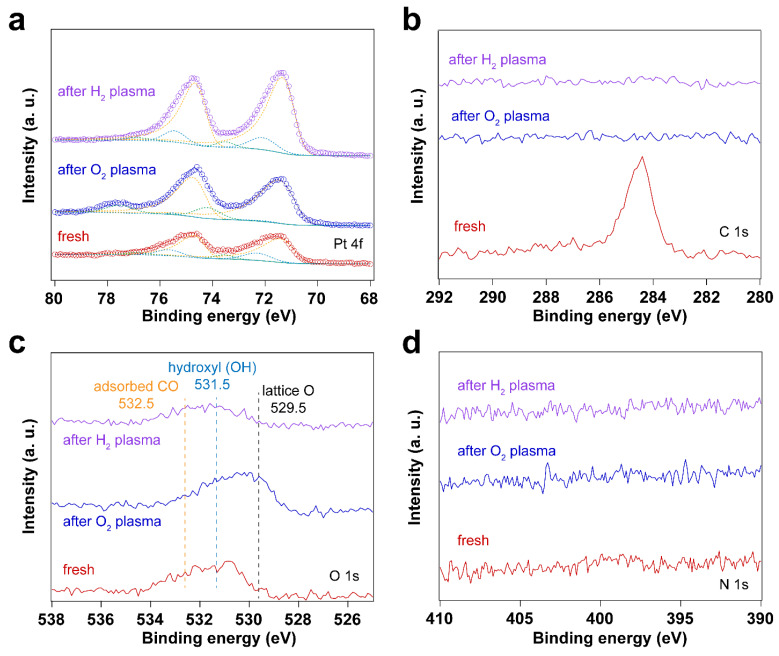
XP spectra of surfactant encapsulated Pt nanoparticles after O_2_ plasma treatment followed by H_2_ plasma reduction processes at room temperature: (**a**) Pt 4f XP spectra; (**b**) C 1s XP spectra; (**c**) O 1s XP spectra; (**d**) N 1s XP spectra. The pressure of O_2_ or H_2_ for plasma treatment was 0.1 mbar, and the power applied for plasma treatment was 4 W.

In addition to surface cleaning, H_2_ plasma can also be used to reduce Pt oxides. The two O_2_-based cleaning methods described before result in the formation of oxides. We explore here the reduction of these oxides in different ways: (1) UV-ozone treatment coupled with in situ thermal H_2_ reduction, and (2) in situ O_2_ plasma followed by H_2_ plasma treatment. Figure 7 shows XPS measurements of surfactant encapsulated Pt nanoparticles after UV-ozone treatment followed by in situ H_2_ reduction at 100 °C under 0.5 mbar H_2_. Table 4 summarizes the Pt 4f peak positions and area ratios for the different Pt species before and after UV-ozone treatment, followed by in situ H_2_ reduction. Figure 8 shows the surfactant encapsulated Pt nanoparticles after O_2_ plasma treatment followed by H_2_ plasma reduction processes at room temperature. The Pt 4f peak positions and area ratios for different Pt species are summarized in Table 5. These results suggest that oxidized Pt can be reduced via thermal or plasma treatment in H_2_. Thermal treatment induces the C species emerging from bulk after O_2_ plasma cleaning, while no trace amount of C was detected after H_2_ plasma treatment. Figure S6 in the supporting information demonstrates that the H_2_ plasma treatment after O_2_ plasma further helps clean the surface. Residual hydrocarbon species desorb from the surface of Pt nanoparticles during H_2_ plasma treatment.

**Figure 9:**
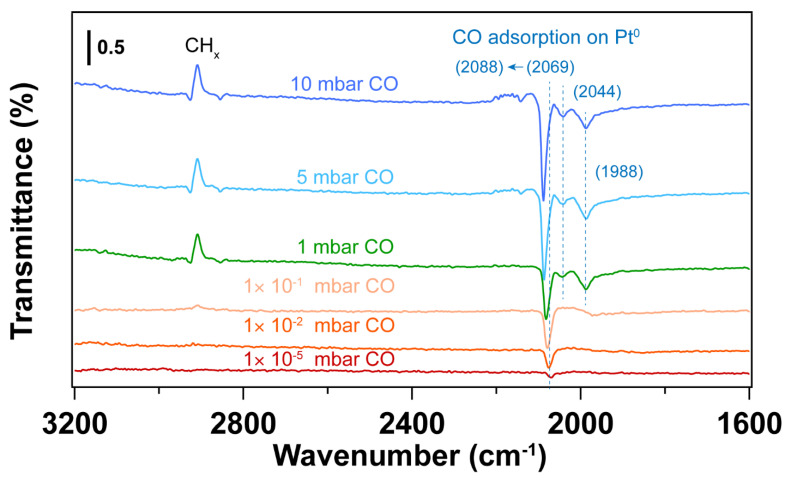
IRRA spectra of in situ CO adsorption on Pt nanoparticles, after UV-ozone followed by thermal H_2_ reduction treatment. The wavenumbers of CO vibration are presented in brackets. Before introducing CO, background ($\frac{p_{0}}{s_{0}}$) was collected under UHV conditions (2 × 10^−8^ mbar). The transmittance signal was obtained by comparing the spectra under different CO pressures with the background spectrum ($\frac{{p_{CO}/s}_{CO}}{{p_{0}/s}_{0}}$), where *p_CO_* and *s_CO_* refer to the *p*- and *s*-polarized spectra that were collected under different CO pressures.

**Figure 10:**
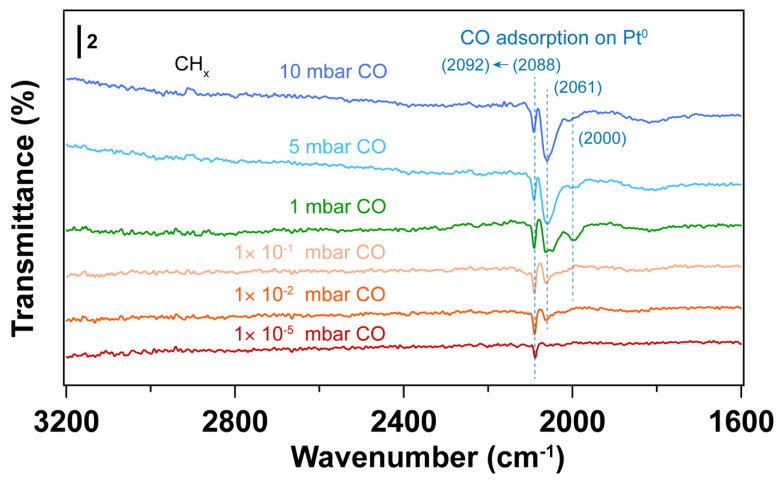
IRRA spectra of in situ CO adsorption on Pt nanoparticles after O_2_ plasma followed by H_2_ plasma treatment. The wavenumbers of CO vibration are presented in brackets. The pressure of O_2_ or H_2_ for plasma treatment was 0.1 mbar, power applied for plasma treatment was 4 W. Before introducing CO, the background ($\frac{p_{0}}{s_{0}}$) was collected under UHV conditions (2 × 10^−8^ mbar). The transmittance signal was obtained by comparing the spectra under different CO pressures with the background spectrum ($\frac{{p_{CO}/s}_{CO}}{{p_{0}/s}_{0}}$); where *p_CO_* and *s_CO_* refer to the *p*- and *s*- polarized spectra that were collected under different CO pressures. In situ tracking of structure changes by IRRAS during sequential plasma treatments are presented in the supporting information.

Figure 9 shows IRRA spectra of in situ CO adsorption on Pt nanoparticles after UV-ozone followed by thermal H_2_ reduction treatment. Figure 10 shows the IRRA spectra of in situ CO adsorption on Pt nanoparticles after O_2_ plasma treatment followed by H_2_ plasma treatment. For both samples, CO adsorption on highly coordinated sites dominates at low pressures (<1 × 10^−1^ mbar). With increase of CO pressure, lower CO vibrational frequencies appear, which is similar to the H_2_ plasma treatment as discussed above (Figure 6). Since H_2_ plasma helps cleaning Pt nanoparticle surface after O_2_ plasma treatment, only a small fraction of hydrocarbon species at 3000–2800 cm^−1^ desorbs from the surface at high CO pressure (5 mbar). In contrast, the thermal reduction of Pt nanoparticles by H_2_ does not help the desorption of hydrocarbon species. During CO adsorption experiments, the desorption of hydrocarbon is observed at a relatively lower CO pressure (1 mbar).

### 3.5. Practical Applications of Plasma Cleaning in Advanced Characterization

**Figure 11:**
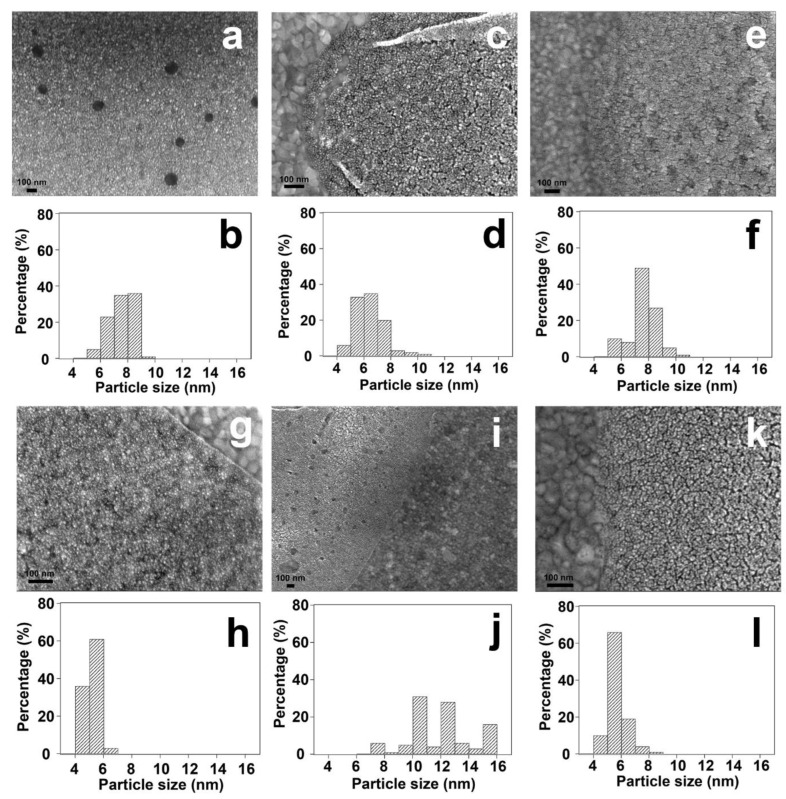
SEM analysis of the changes of surfactant encapsulated Pt nanoparticles before and after treatments. (**a**,**b**) surfactant encapsulated Pt nanoparticles supported on Au film without treatment; (**c**,**d**) surfactant encapsulated Pt nanoparticles supported on Au film after UV-ozone treatment; (**e**,**f**) surfactant encapsulated Pt nanoparticles supported on Au film after O_2_ plasma treatment; (**g**,**h**) surfactant encapsulated Pt nanoparticles supported on Au film after H_2_ plasma treatment; (**i**,**j**) surfactant encapsulated Pt nanoparticles supported on Au film after UV-ozone treatment followed by H_2_ reduction at 100 °C for 30 min; (**k**,**l**) surfactant encapsulated Pt nanoparticles supported on Au film after O_2_ plasma followed by H_2_ plasma treatment. The estimated particle size distributions were obtained by counting ~100 nanoparticles from the SEM images.

To further investigate the changes of particle size after various treatments, scanning electron microscopy (SEM) analysis is performed. As shown in Figure 11 and Figures S9–S13 in supporting information, the average particle size of Pt nanoparticles is ~7.5 nm for the fresh sample. Non-thermal treatments (UV-ozone and plasma treatments) do not influence average particle size significantly, while thermal reduction leads to the growth of average Pt nanoparticle size. As shown in Figure 11j, a broad particle size distribution of Pt is observed after thermal reduction treatment. The presence of large particles (>12 nm) indicates the agglomeration of Pt nanoparticles during thermal treatment, which is consistent with the atomic force microscopy (AFM) result as shown in Figure S16. Compared to the thermal treatment, Pt nanoparticles show narrower size distributions after non-thermal treatments. In addition, O_2_-based surface cleaning treatment (UV-ozone and O_2_ plasma) results in the formation of Pt aggregates containing several small nanoparticles. On the contrary, the dispersion of Pt nanoparticles remains almost unchanged after H_2_ plasma treatment. As shown in Figure S17, there is no obvious agglomeration of Pt nanoparticles after H_2_ plasma treatment. These results indicate that compared to O_2_-based surface cleaning methods, H_2_ plasma treatment can efficiently remove hydrocarbon species without significantly changing the structure of nanoparticles.

**Figure 12:**
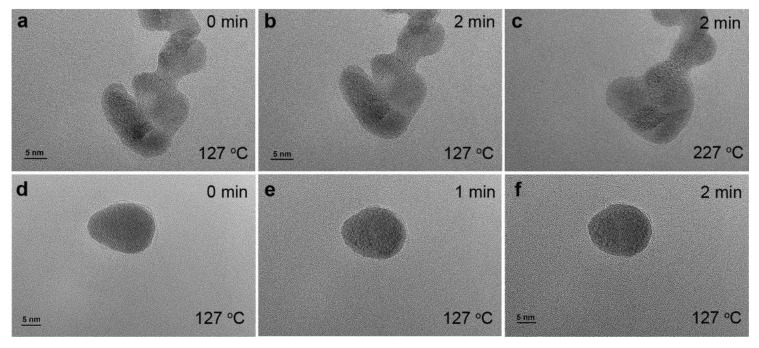
(**a**–**c**) In situ ETEM images showing morphological evolution of encapsulated Pt nanoparticles as a function of time and temperature; (**d**–**f**) in situ ETEM images of Pt nanoparticle after in situ air plasma treatment for 1 h at 127 °C. The shape changes of different nanoparticles are shown in Movies S1–S3 in the supporting information.

Collectively, the widely used surfactant in chemical synthesis helps prepare metal nanoparticles with small particle sizes, which may exhibit promising performances due to the size effect, the formation of high density of defective sites and the different facets with atomic arrangements. However, after synthesis, the residual surfactant molecules on metal nanoparticle surface can either work cooperatively with metal nanoparticles to generate novel properties or block the surface of metal nanoparticles, sacrificing the surface metal sites. As shown in Figure S18, the presence of surfactant occupies surface Pt sites and thus the absence of CO adsorption on Pt was observed at low CO pressures. Both non-thermal UV-ozone and plasma treatment show the efficient removal of surfactant, indicated by the strong CO adsorption on Pt nanoparticles. As an example, Figure 12 compares the shape changes of Pt nanoparticles by conducting environmental transmission electron microscopy (ETEM) under different conditions to show the advantage of plasma-based cleaning method in practical applications. Figure 12a,b shows that surfactant encapsulated Pt nanoparticles are stable at 127 °C. The shapes of these particles remain almost unchanged after 2 min under vacuum conditions (~1 × 10^−6^ mbar). Further increasing the temperature to 227 °C, the small particles merge together, leading to the shape changes of these nanoparticles, as shown in Figure 12c. Movies S1 and S2 in the supporting information also show highly mobile Pt and shape changes of Pt nanoparticles at 227 °C. However, this dynamic structural reconstruction is absent at 127 °C. Figure 12d–f shows that after air plasma treatment for 1 h, the shape and size of Pt nanoparticles changes in 1 min. Longer exposing time results in the further changes in shape and particle size. Movie S3 in the supporting information also indicates that the structure undergoes reconstruction at 127 °C under vacuum conditions, after plasma treatment. The utilization of air plasma is more complicated than the single gas induced plasma treatment due to the complicated gas composition. Typically, reactive oxygen and nitrogen species, such as ozone, active radicals, nitrogen oxides, etc., play a major role in air plasma [55]. These results indicate the efficient sample cleaning by non-thermal plasma treatment.

## 4. Conclusions

In this work, we have investigated different strategies for the removal of surfactants from metal surfaces using well-defined Pt nanoparticles as model systems. The removal methods include UV-ozone irradiation, and non-thermal O_2_ and H_2_ plasma treatments. XPS, IRRAS analyses coupled with CO probe experiments demonstrate the efficient surfactant removal by all methods. For O_2_-based cleaning methods, surface Pt oxides are formed after treatment, and the particle size of Pt increases. On the contrary, H_2_ plasma treatment prevents oxidation and agglomeration of Pt nanoparticles. It also leads to a cleaner surface without residual adsorbed hydrocarbons after treatment. These observations reveal the importance of treatment conditions in producing a clean surface of nanoparticles without significant changes in structure and properties. Environmental TEM shows the highly dynamic surface of Pt nanoparticles after plasma treatment, indicating the protective role of surfactant and the efficient removal of surfactant by plasma treatment. SEM analysis demonstrates that the particle size distribution remains almost unchanged after non-thermal H_2_ plasma treatment. Notably, the dispersion of Pt nanoparticles is unchanged by H_2_ plasma treatment. While O_2_-based treatments do not lead to the growth of Pt nanoparticles, they induce changes in the dispersion of Pt nanoparticles, forming Pt aggregates. We hope these findings will open various opportunities for development of surface cleaning by extension of the concept to other materials and surfactants as well as gases used in plasma treatment. Note that for more electropositive metals than Pt, which are sensitive to oxidizing conditions, the utilization of non-thermal H_2_ plasma may be the only possible option to prevent drastic oxidation of the metal.

## Figures and Tables

**Table 1:** Pt 4f peak positions and area ratios for the different Pt species before and after UV-ozone treatment, and CO adsorption experiment.

Sample		Fresh	After UV-Ozone Treatment	After CO Adsorption
Peak position (eV)	Pt^0^ 4f_7/2_	71.17	71.16	71.04
	Pt^0^ 4f_5/2_	74.52	74.51	74.39
	Pt–O(I) 4f_7/2_	72.45	72.70	72.18
	Pt–O(I) 4f_5/2_	75.80	76.05	75.53
	Pt–O(II) 4f_7/2_	73.79	73.98	73.57
	Pt–O(II) 4f_5/2_	77.14	77.33	76.92
Peak area ratio	Pt–O(I)/Pt^0^	0.28	0.13	0.30
	Pt–O(II)/Pt^0^	0.07	0.37	0.27

**Table 2:** Pt 4f peak positions and area ratios for the different Pt species before and after O_2_ plasma treatment and in situ CO adsorption.

Sample		Fresh	After O_2_ Plasma Treatment	After CO Adsorption
Peak position (eV)	Pt^0^ 4f_7/2_	71.16	71.05	71.07
	Pt^0^ 4f_5/2_	74.51	74.40	74.42
	Pt–O(I) 4f_7/2_	73.69	73.06	73.43
	Pt–O(I) 4f_5/2_	77.04	76.41	76.77
	Pt–O(II) 4f_7/2_	72.48	74.29	74.80
	Pt–O(II) 4f_5/2_	75.83	77.64	78.15
Peak area ratio	Pt–O(I)/Pt^0^	0.13	0.04	0.04
	Pt–O(II)/Pt^0^	0.07	0.17	<0.001

**Table 3:** Pt 4f peak positions and area ratios for the different Pt species before and after H_2_ plasma treatment and in situ CO adsorption.

Sample		Fresh	After H_2_ Plasma Treatment	After CO Adsorption
Peak position (eV)	Pt^0^ 4f_7/2_	71.22	71.05	71.07
	Pt^0^ 4f_5/2_	74.57	74.40	74.42
	Pt–O(I) 4f_7/2_	72.50	72.05	72.46
	Pt–O(I) 4f_5/2_	75.85	75.40	75.81
	Pt–O(II) 4f_7/2_	74.60	73.58	73.54
	Pt–O(II) 4f_5/2_	77.95	76.93	76.89
Peak area ratio	Pt–O(I)/Pt^0^	0.15	0.03	0.03
	Pt–O(II)/Pt^0^	0.08	0.02	0.02

**Table 4:** Pt 4f peak positions and area ratios for the different Pt species before and after UV-ozone treatment followed by in situ H_2_ reduction.

Sample		Fresh	After UV-Ozone Treatment	After H_2_ Reduction
Peak position (eV)	Pt^0^ 4f_7/2_	71.14	71.13	71.02
	Pt^0^ 4f_5/2_	74.49	74.48	74.37
	Pt–O(I) 4f_7/2_	72.47	72.70	72.58
	Pt–O(I) 4f_5/2_	75.82	76.05	75.93
	Pt–O(II) 4f_7/2_	73.72	73.88	73.54
	Pt–O(II) 4f_5/2_	77.07	77.23	76.89
Peak area ratio	Pt–O(I)/Pt^0^	0.15	0.07	0.02
	Pt–O(II)/Pt^0^	0.09	0.30	0.04

**Table 5:** Pt 4f peak positions and area ratios for the different Pt species before and after O_2_ plasma treatment followed by H_2_ plasma treatment.

Sample		Fresh	After O_2_ Plasma Treatment	After H_2_ Plasma Treatment
Peak position (eV)	Pt^0^ 4f_7/2_	71.07	71.08	71.03
	Pt^0^ 4f_5/2_	74.42	74.43	74.38
	Pt–O(I) 4f_7/2_	72.31	72.15	72.10
	Pt–O(I) 4f_5/2_	75.66	75.50	75.45
	Pt–O(II) 4f_7/2_	73.56	74.19	73.52
	Pt–O(II) 4f_5/2_	76.91	77.54	76.87
Peak area ratio	Pt–O(I)/Pt^0^	0.22	0.01	0.13
	Pt–O(II)/Pt^0^	0.08	0.15	0.04

## Data Availability

Data are contained within the article and supplementary materials.

## Supplementary Materials

The following supporting information can be downloaded at: https://www.mdpi.com/article/10.3390/nano14030290/s1. Reference [56] is cited in the supplementary materials.
