# Supplementary material for: In Situ Monitoring of Non-Thermal Plasma Cleaning of Surfactant Encapsulated Nanoparticles
Source: Nanomaterials (Basel). 2024 Jan 31;14(3):290. doi: 10.3390/nano14030290 (PMC10856489; doi:10.3390/nano14030290)
Supplement: Supplementary file 1 [file nanomaterials-14-00290-s001.zip › Supplementary material.pdf]

`*Supporting Information*

In Situ Monitoring of Non-Thermal Plasma Cleaning of Surfactant Encapsulated Nanoparticles

Gengnan Li [1,*], Dmitri N. Zakharov [1], Sayantani Sikder [1,2], Yixin Xu [1,2], Xiao Tong [1], Panagiotis Dimitrakellis [3] and Jorge Anibal Boscoboinik [1,*]

[1] Center for Functional Nanomaterials, Brookhaven National Laboratory, Upton, New York 11973, United States

[2] Department of Materials Science and Chemical Engineering, Stony Brook University, Stony Brook, New York 11790, United States

[3] Catalysis Center for Energy Innovation, University of Delaware, Newark, Delaware 19716, United States; Department of Chemical and Biomolecular Engineering, University of Delaware, Newark, Delaware 19716, United States

*Corresponding author: G. Li (gli2@bnl.gov); J.A. Boscoboinik (E-mail: jboscoboinik@bnl.gov)

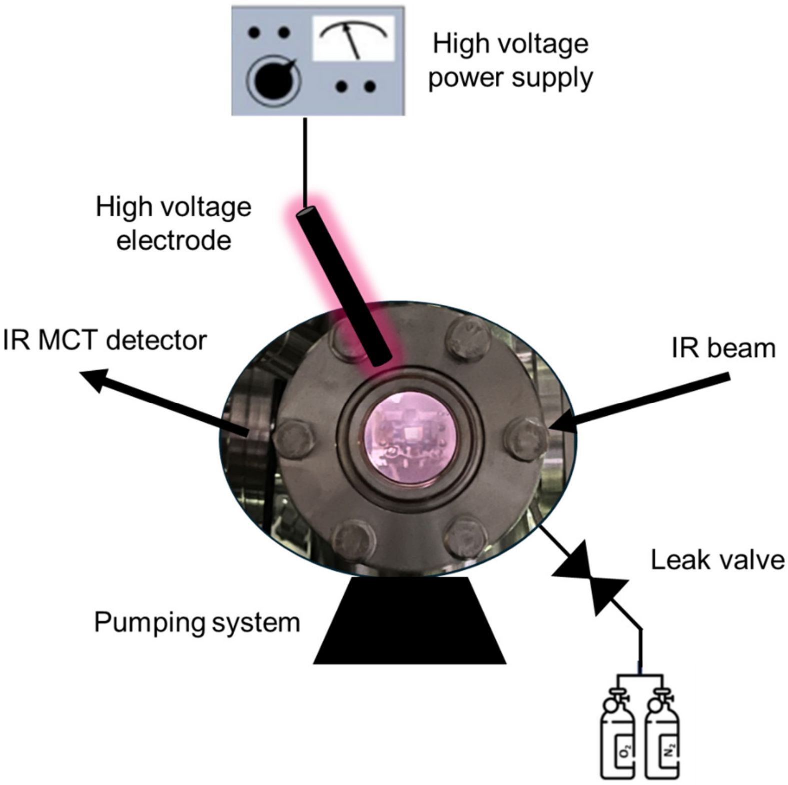

Scheme S1. Plasma setup in IRRAS.

Ex situ UV-ozone treatment

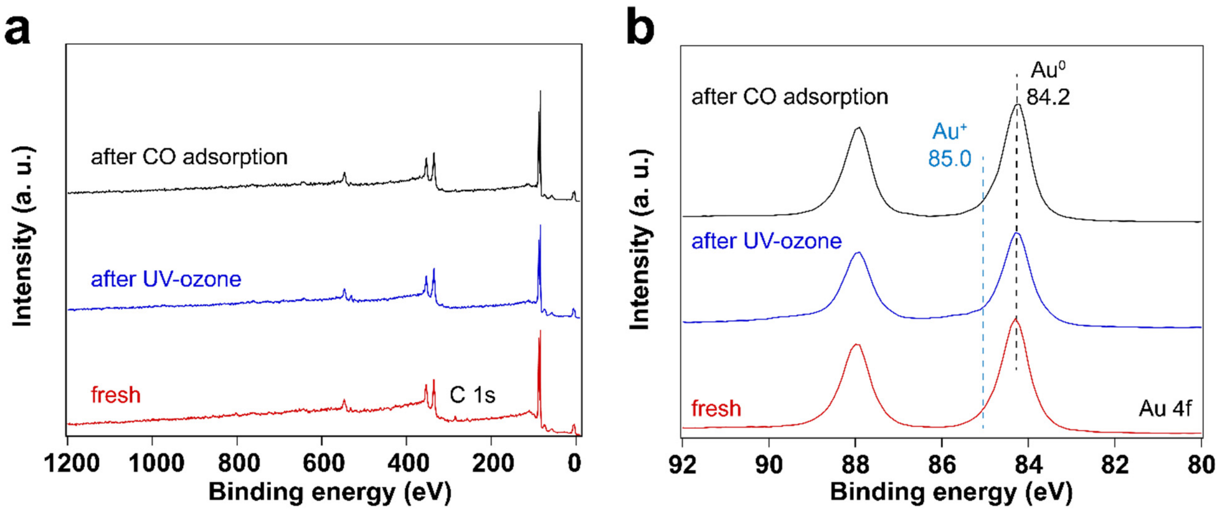

Figure S1. (a) Survey spectra and (b) Au 4f XP spectra of surfactant encapsulated Pt nanoparticles before and after UV-ozone treatment and after in situ CO adsorption experiment.

Table S1. Surface composition of Pt nanoparticles deposited on Au film derived from high-resolution XPS spectra.

Area ratio	C1s/Au4f	O1s/Au4f	N1s/Pt4f	Pt4f/Au4f
**Fresh sample**	0.83	0.27	0.17	0.08
**After UV-ozone treatment**	0.12	0.35	-	0.09
**After CO adsorption experiment**	0.02	0.13	-	0.05

In situ CO adsorption on Pt nanoparticles after UV-ozone treatment

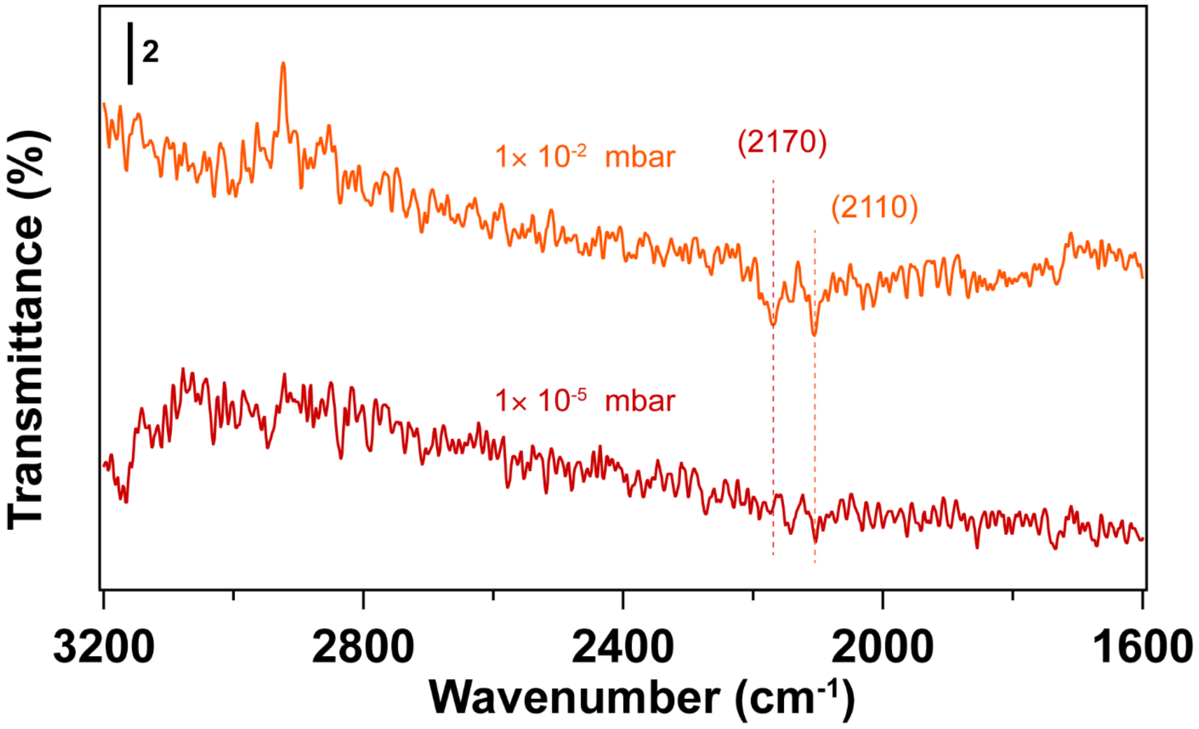

Figure S2. IRRAS spectra of in situ CO adsorption on surfactant encapsulated Pt nanoparticles after UV-ozone treatment. It shows CO adsorption on Pt-O sites with vibrational frequencies of 2170 and 2110 cm^{-1}.

In situ O_2 plasma treatment

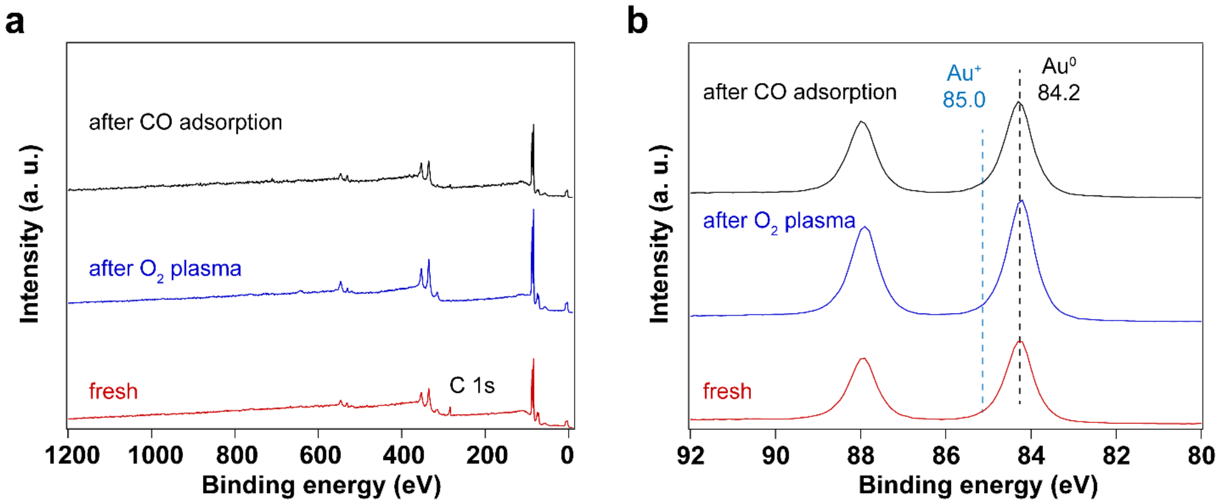

Figure S3. (a) Survey spectra and (b) Au 4f XP spectra of surfactant encapsulated Pt nanoparticles before and after O_2 plasma treatment.

Table S2. Surface composition of Pt nanoparticles deposited on Au film derived from high-resolution XPS spectra.

Area ratio	C1s/Au4f	O1s/Au4f	N1s/Pt4f	Pt4f/Au4f
**Fresh sample**	1.83	0.50	0.16	0.30
**After O_2 plasma treatment**	0.01	0.29	0.01	0.25

In situ H_2 plasma treatment

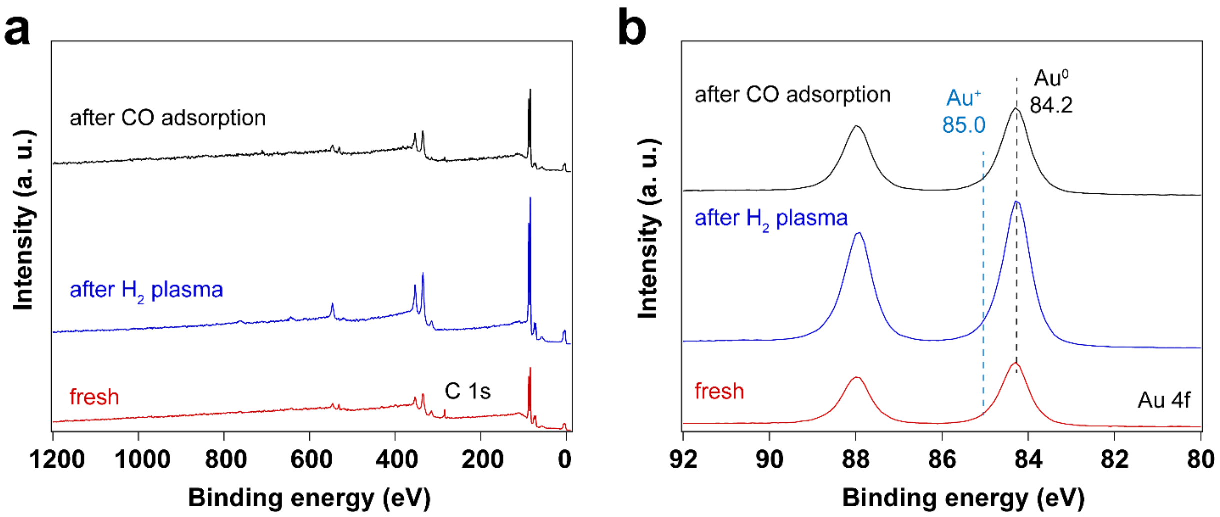

Figure S4. (a) Survey spectra and (b) Au 4f XP spectra of surfactant encapsulated Pt nanoparticles before and after H_2 plasma treatment.

Table S3. Surface composition of Pt nanoparticles deposited on Au film derived from high-resolution XPS spectra.

Area ratio	C1s/Au4f	O1s/Au4f	N1s/Pt4f	Pt4f/Au4f
**Fresh sample**	2.08	0.52	0.43	0.31
**After H_2 plasma treatment**	0.86	0.06	0.06	0.18

Ex situ UV-ozone treatment followed by in situ H_2 reduction at 100 °C

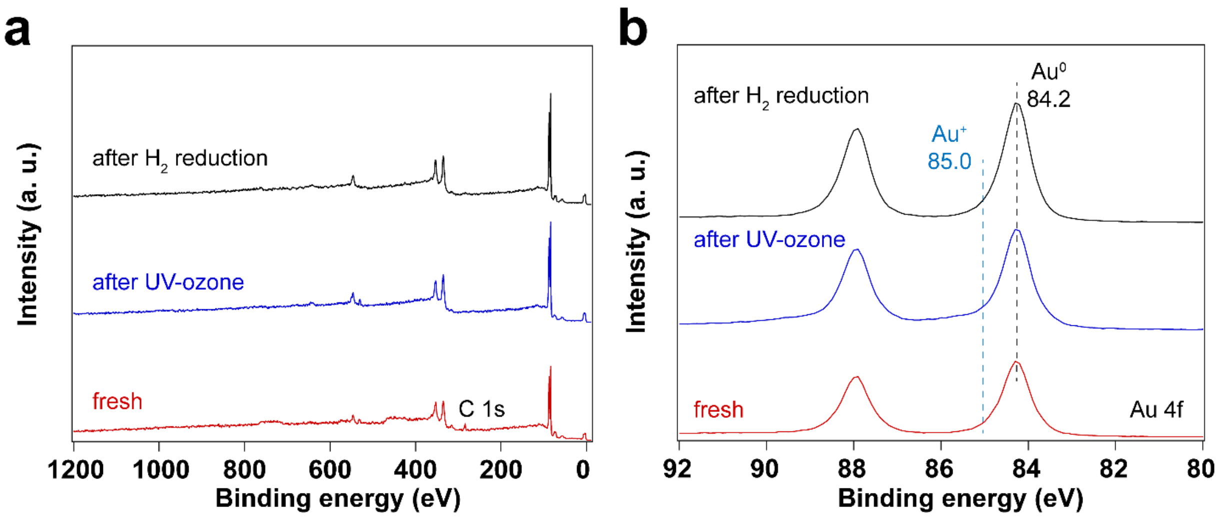

Figure S5. (a) Survey spectra and (b) Au 4f XP spectra of surfactant encapsulated Pt nanoparticles before and after UV-ozone and H_2 plasma treatment.

Table S4. Surface composition of Pt nanoparticles deposited on Au film derived from high-resolution XPS spectra.

Area ratio	C1s/Au4f	O1s/Au4f	N1s/Pt4f	Pt4f/Au4f
**Fresh sample**	1.25	0.37	0.17	0.11
**After UV-ozone treatment**	0.13	0.30	-	0.07
**After H_2 reduction**	0.20	0.06	0.05	0.07

In situ O_2 plasma followed by H_2 plasma treatment

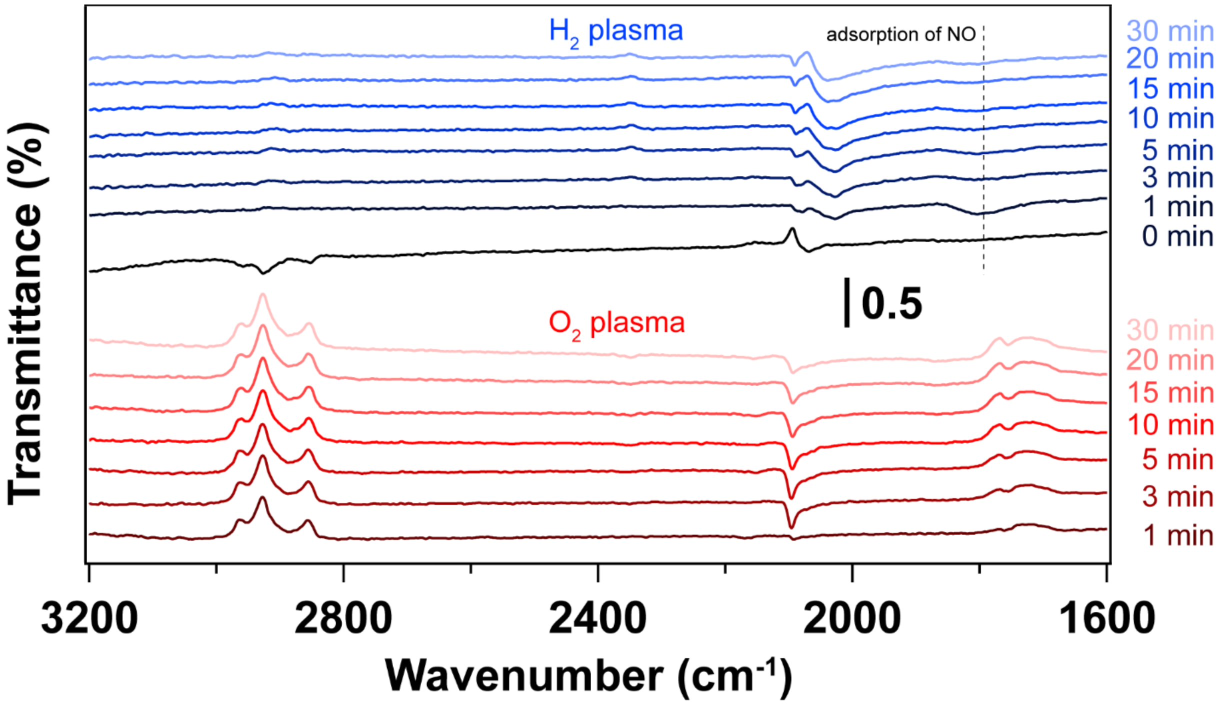

Figure S6. IRRAS spectra of surfactant encapsulated Pt nanoparticles by sequential O_2-H_2 plasma treatment. The pressure of O_2 or H_2 was 0.1 mbar, power applied for plasma treatment was 4 W.

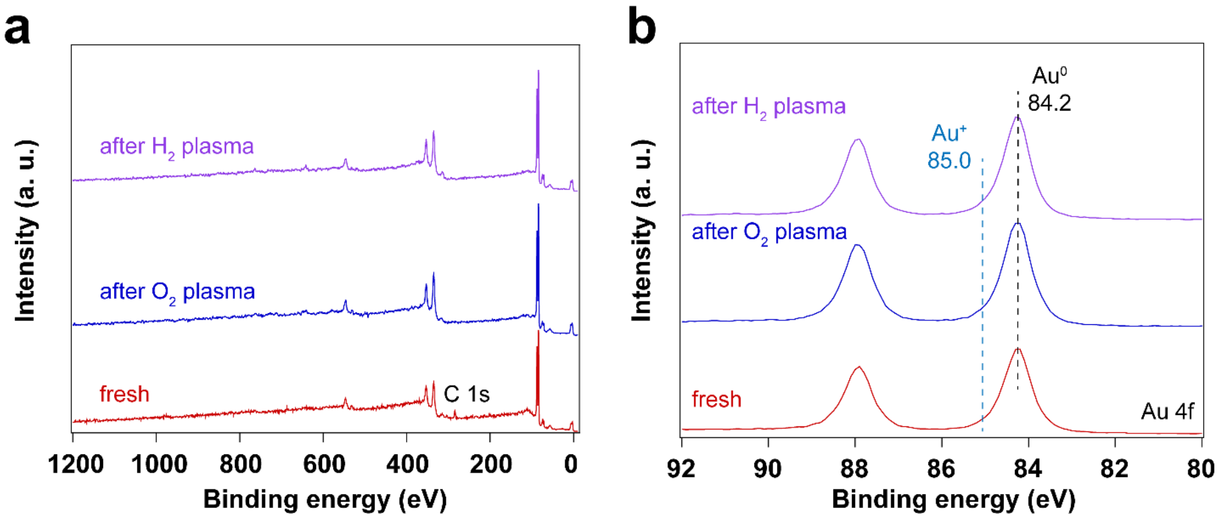

Figure S7. (a) Survey spectra and (b) Au 4f XP spectra of surfactant encapsulated Pt nanoparticles before and after O_2 and H_2 plasma treatment.

Table S5. Surface composition of Pt nanoparticles deposited on Au film derived from high-resolution XPS spectra.

Area ratio	C1s/Au4f	O1s/Au4f	N1s/Pt4f	Pt4f/Au4f
**Fresh sample**	1.33	0.26	0.14	0.08
**After O_2 plasma treatment**	0.03	0.26	-	0.11
**After H_2 plasma treatment**	0.07	0.12	-	0.16

Scanning electron microcopy analysis of Pt nanoparticles on Au substrate

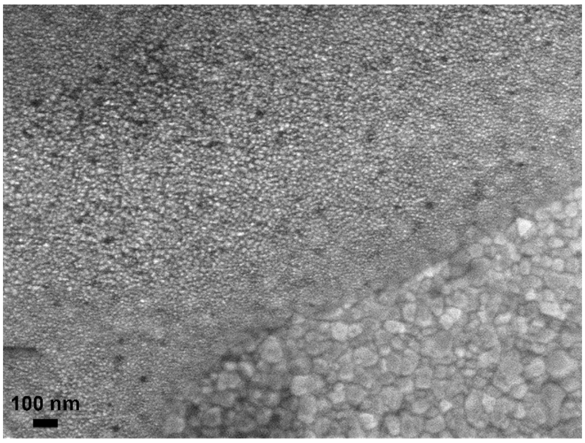

Figure S8. SEM image of fresh surfactant encapsulated Pt nanoparticles on Au film.

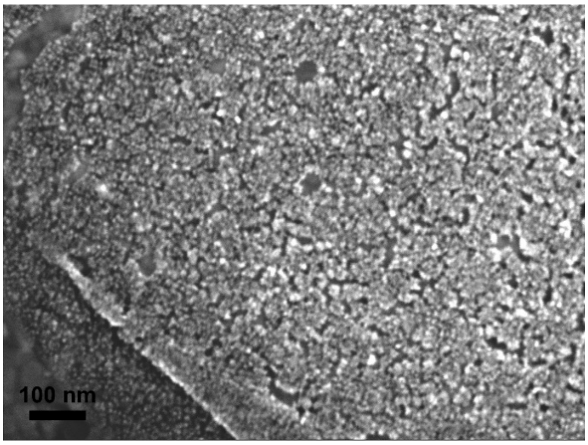

Figure S9. SEM image of surfactant encapsulated Pt nanoparticles on Au film after UV-ozone treatment.

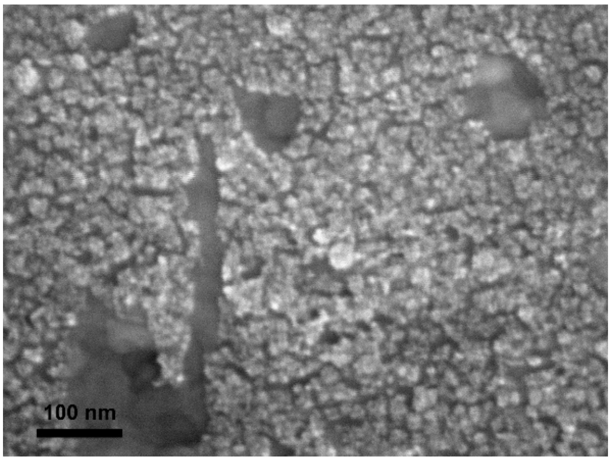

Figure S10. SEM image of surfactant encapsulated Pt nanoparticles on Au film after UV-ozone treatment followed by H_2 reduction at 100 °C.

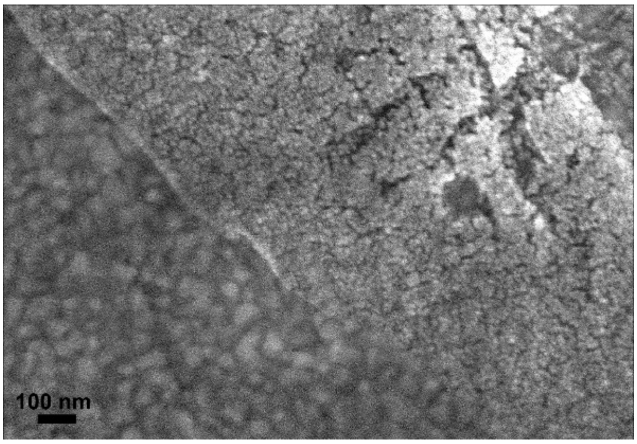

Figure S11. SEM image of surfactant encapsulated Pt nanoparticles on Au film after O_2 plasma treatment.

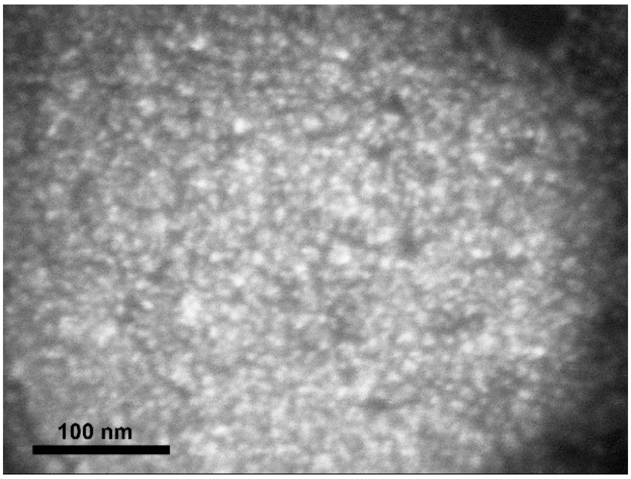

Figure S12. SEM image of surfactant encapsulated Pt nanoparticles on Au film after H_2 plasma treatment.

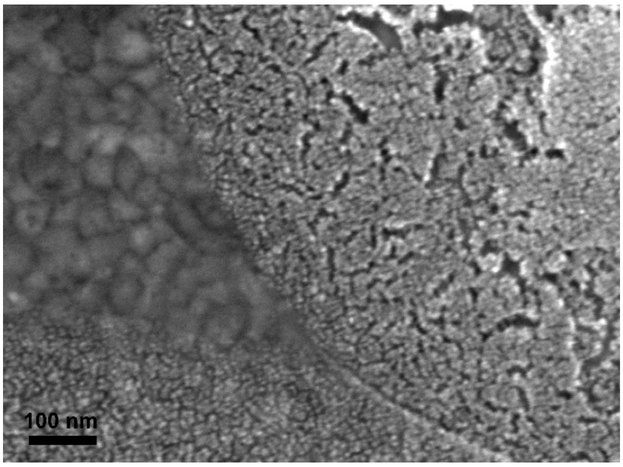

Figure S13. SEM image of surfactant encapsulated Pt nanoparticles on Au film after O_2 plasma treatment followed by H_2 plasma treatment.

Atomic force microcopy analysis of Pt nanoparticles on Au substrate

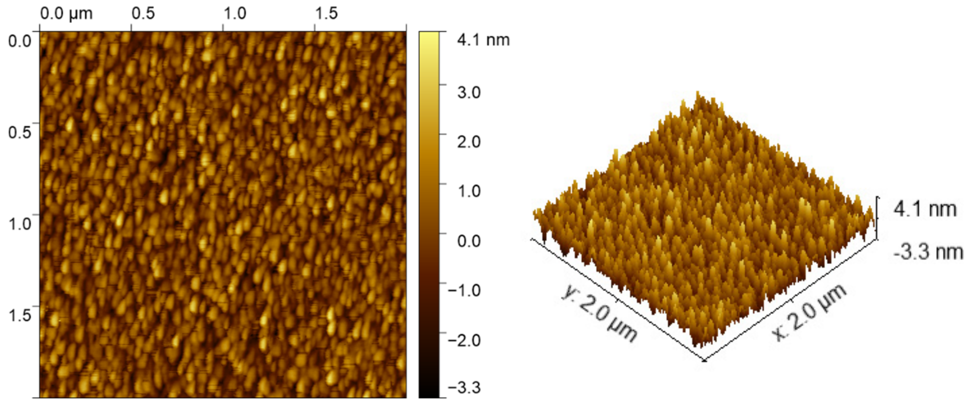

Figure S14. Atomic force microscopy (AFM) image of Au substrate.

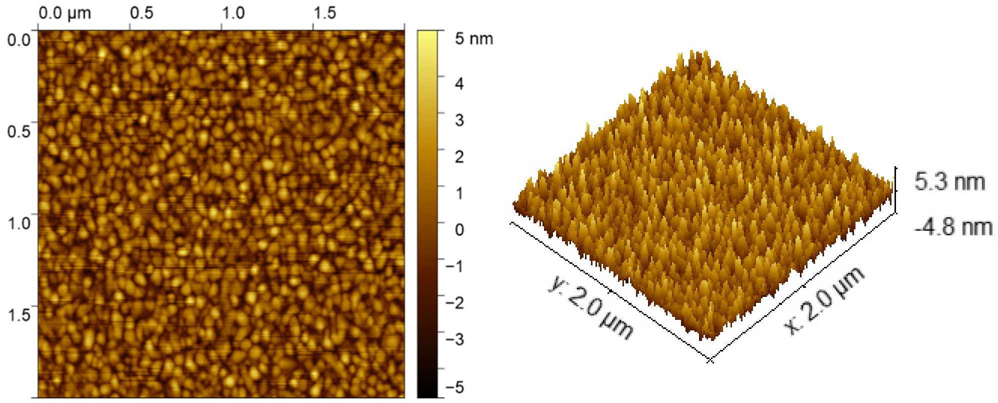

Figure S15. Atomic force microscopy (AFM) image of surfactant encapsulated Pt nanoparticles on Au substrate.

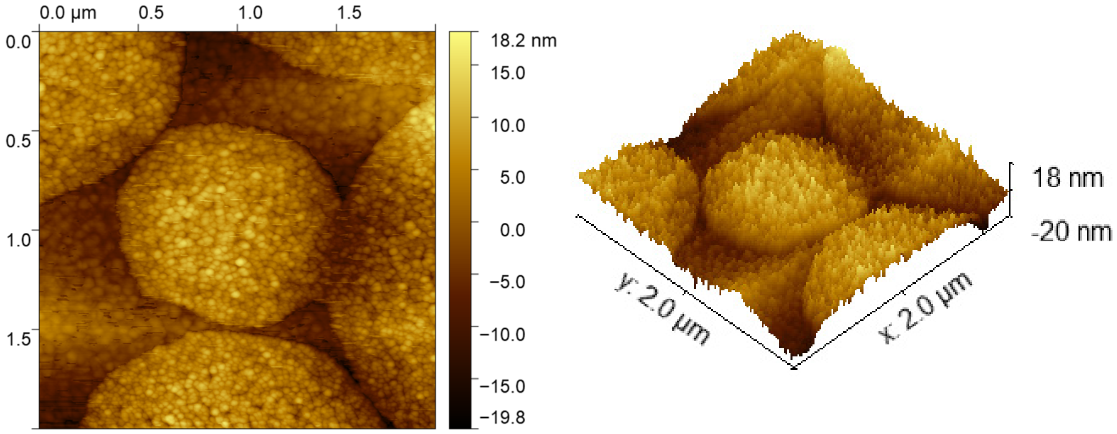

Figure S16. Atomic force microscopy (AFM) image of surfactant encapsulated Pt nanoparticles on Au substrate after thermal treatment under O_2.

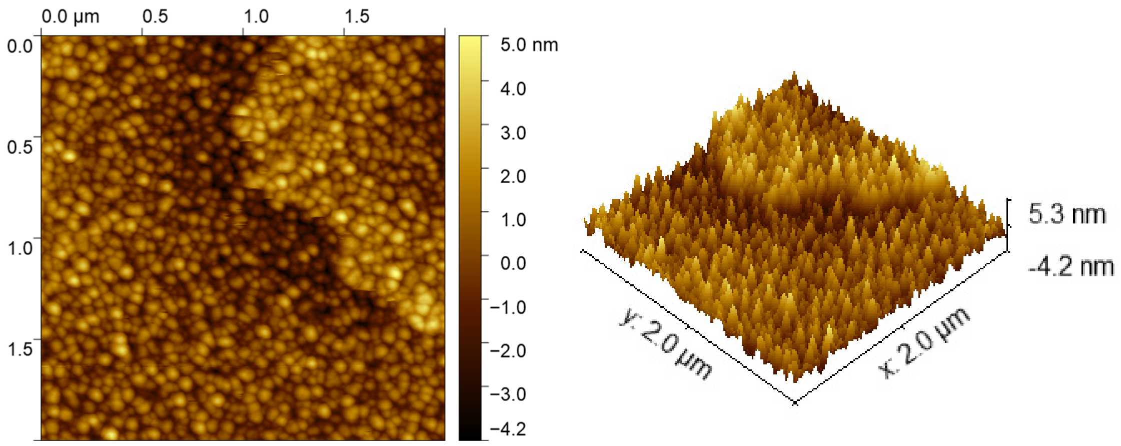

Figure S17. Atomic force microscopy (AFM) image of surfactant encapsulated Pt nanoparticles on Au substrate after H_2 plasma treatment.

In situ CO adsorption on surfactant encapsulated Pt nanoparticles

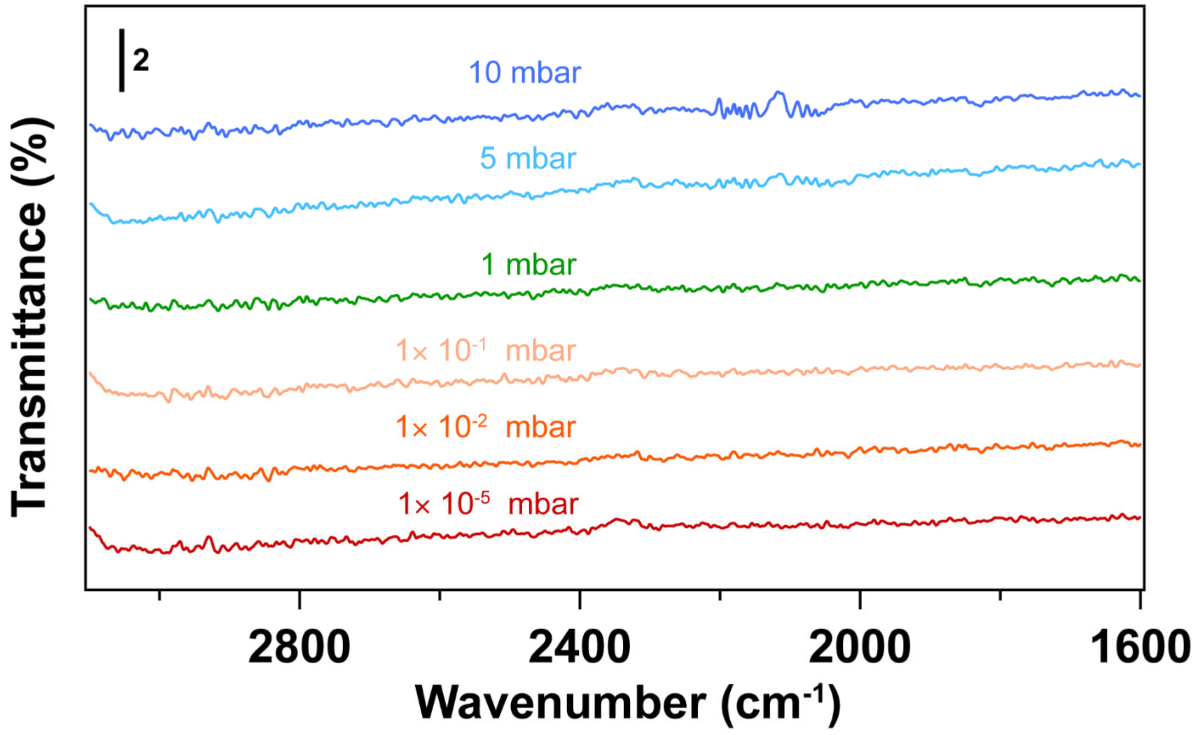

Figure S18. IRRA spectra of in situ CO adsorption on surfactant encapsulated Pt nanoparticles.

Optical emission spectroscopy (OES) in H_2 plasma

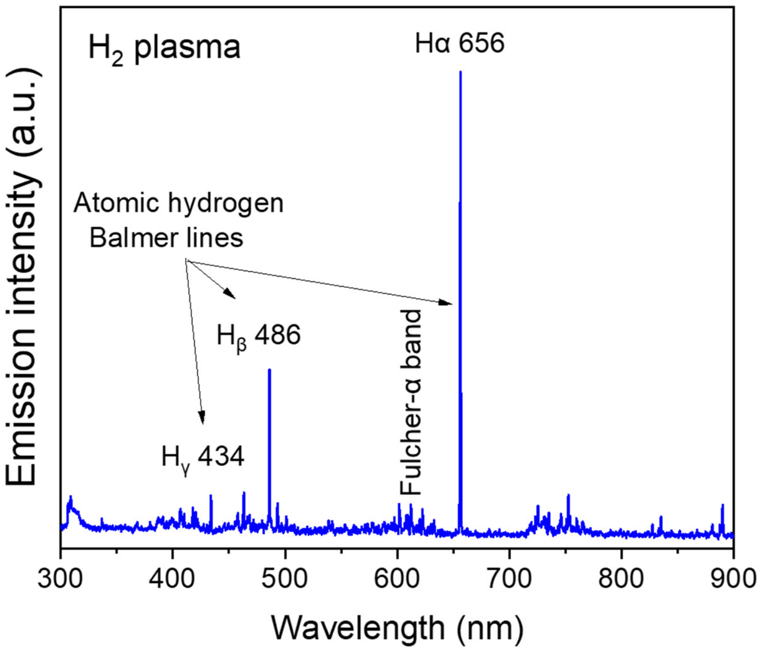

Figure S19. Optical emission spectrum of H_2 plasma used for the removal of surfactants from Pt nanoparticles. The atomic H Balmer lines as well as the Fulcher-a band are dominating the emission spectrum.[1]

[1] N. Škoro, N. Puač, S. Lazović, U. Cvelbar, G. Kokkoris, E. Gogolides, Characterization and global modelling of low-pressure hydrogen-based RF plasmas suitable for surface cleaning processes, Journal of Physics D: Applied Physics, 46 (2013) 475206.
